# Mule trains to mountain roads: the role of working mules in supporting resilient communities in the Himalayas

**DOI:** 10.3389/fvets.2024.1390644

**Published:** 2024-07-31

**Authors:** Laura M. Kubasiewicz, Tamlin Watson, Sajana Thapa, Caroline Nye, Natasha Chamberlain

**Affiliations:** ^1^Equine Operations, The Donkey Sanctuary, Sidmouth, United Kingdom; ^2^Animal Nepal, Lalitpur, Nepal; ^3^Centre for Rural Policy Research, University of Exeter, Exeter, United Kingdom

**Keywords:** equid welfare, human development, one welfare, remote communities, sustainable development goals

## Abstract

Working equids play a central role in mountainous communities, but their work often goes unnoticed by the wider world, with sparse documentation of their role, value, or welfare – a state which often extends to their human counterparts. Communities living in the remote Manaslu Valley, Nepal, face a number of uncertainties, including extreme weather events due to the seasonal monsoon and, more recently, the construction of a new road network. Using semi-structured interviews, questionnaires, and Equine Assessment Research and Scoping (EARS) welfare assessments, we outline the specific role of pack mules in supporting the lives of local people, explain the nuanced links between human experience and mule welfare, and gain insight into how people living in this volatile environment manage uncertainty and risk. Mule work was felt to be the ‘only option’ for a sustainable livelihood for most mule owners although, in some cases, mules had enabled respondents to diversify their income. Mule owners with more husbandry experience did not own mules in more positive behavioural states, which may suggest a lack of generational knowledge and support networks. Short-term ongoing risks, such as the monsoon or unstable tracks, had a larger impact both financially and emotionally than the long-term but distant implications of the road construction. Mule owners must constantly balance the risk of working during the monsoon season, when conditions are treacherous but pay was higher, with losing valuable income but keeping themselves and their mules safe; they do, however, have a more mobile option for employment than non-owners. Mules enable a level of resilience and agility for communities living with constant uncertainty and change, which is only beginning to be recognised formally within the sustainable development sphere. Integration of animal welfare into the SDGs would allow humanitarian aid initiatives to strengthen support networks around working equids, which would greatly benefit the mules and humans alike.

## 1 Introduction

In remote communities, working equids are often a fundamental part of life, enabling people to access support or resources that would otherwise be difficult to reach (1, 2). For example, donkeys (*Equus asinus*) and mules (*E. asinus × E. caballus*) transport firewood and grain for rural communities in Ethiopia (3); help Masaai women in Kenya to fetch water (4), and carry produce to market from rural smallholder farms in Nigeria (5). In mountainous areas, access often involves steep, uneven tracks that can only be navigated on foot or by pack animals. In these regions, the support provided by pack mules may extend to direct support for human livelihoods, where opportunities for work would otherwise be limited by both physical access to community hubs and a scarcity of the essential resources required to sustain a business. However, recognition of the role of working equids in human development, and particularly in supporting the United Nations’ Agenda 2030 Sustainable Development Goals (SDGs) has only recently begun to gain traction (6, 7), where working equids are recognised to “*uniquely contribute to the development of more resilient communities*” (8).

Many mountain dwelling communities are sustained in large part by the tourist industry, with people drawn from across the globe to hike and climb in renowned tourist destinations such as the Himalayas. Mules have long supported this industry, and their welfare is closely intertwined with the wellbeing of their human counterparts (9). The Manaslu Valley, Nepal, is a popular trekking destination, where the main trekking route passes through several small communities that offer teahouses for rest, food and accommodation. Whilst mules and their owners work throughout these mountains, transporting ‘food, fuel and firewood’ (10), there is sparse documentation detailing the ways in which they support human livelihoods, or the lived experience of those who work with them. Access to resources in the mountains is as limited for mules as it is for people, where welfare issues are compounded by limited veterinary services (11). In a previous assessment of mule welfare in the region (11), we found that although body condition was generally good, their management was largely inappropriate, characterised by inhumane handling and integumentary trauma from incorrect equipment use. Against this backdrop, we have yet to examine mule ownership from a sociological perspective to assess whether the conditions surrounding mule ownership, such as levels of experience and knowledge transfer to new owners, account for the mules’ substandard welfare.

As with much of the mountainous region of Nepal, the Manaslu Valley is a volatile environment. The region is subject to a seasonal monsoon, which can have a devastating impact on communities living in the steep mountain valleys. The monsoon season, which typically lasts from June to September, brings approximately 1,500–2,500 mm of rain to the Manaslu Conservation Area. Whilst cultivated land in Nepal relies on monsoon precipitation, the intensity of this rainfall frequently results in flooding and landslides which cause damage to infrastructure, loss of life and displacement for millions of people throughout the country (12, 13). Climate change has had an increasing impact on the monsoon, with reports of more extreme and unpredictable weather events and more intense periods of precipitation. Remote communities are particularly vulnerable to these changes, with people living in the Jumla district of Nepal having experienced resource degradation, food scarcity and increased social inequality as a result of the unpredictable weather patterns (14).

In addition to the seasonal risks experienced due to the monsoon, those living in the Manaslu Valley are currently facing a potentially permanent shift in their way of life triggered by the construction of a road through the valley. Expansion of the Nepalese road network, particularly into remote settlements, is a major development goal outlined in the Nepalese constitution of 2015 with the addition of over 7,500 km of paved, dirt and gravel roads in 2017–2018 alone (15). While the road construction project holds the promise of improved economic opportunities and access to essential services, it also heralds the potential to drastically change the environmental and socio-cultural fabric of the valley. In the Andean mountains of Bolivia, numbers of pack donkeys, along with horses and llamas, were greatly reduced following the expansion of road networks into the area (16), although small numbers of donkeys were kept by most families for smaller transport jobs, or for emergency transport when weather conditions made the roads impassable to vehicles (4). Previous road network expansions in Nepal have resulted in excessive logging and the exploitation of natural resources and non-timber forest products, driven by increased access to markets (14). In the hill regions around Dipayal, prior to the availability of alternative heating sources, this exploitation resulted in journeys of several hours for local women to collect firewood, along with an increase in pollution and sanitation problems due to a lack of infrastructure to support the rapidly expanding population brought about by increased access to previously isolated settlements (17).

While multiple definitions of resilience exist, most cover the “*capacity to respond, withstand and/or successfully deal with change, whether at the individual level, community level, state level, or at the level of socio-ecological systems*” (8). The situation in the Manaslu Valley is on the precipice of change, but how the lives of the communities and the mules they work with will shift alongside those changes is largely unknown. We outline the role of mules in supporting resilient communities in the remote mountains and identify the role of mules in meeting the SDGs; explore the relationships between equid handling experience and equid welfare; and provide insight into the mindset of key informants in the face of both current risk exposure, and long-term systemic change from development.

## 2 Methods

### 2.1 Study location

**Figure 1:**
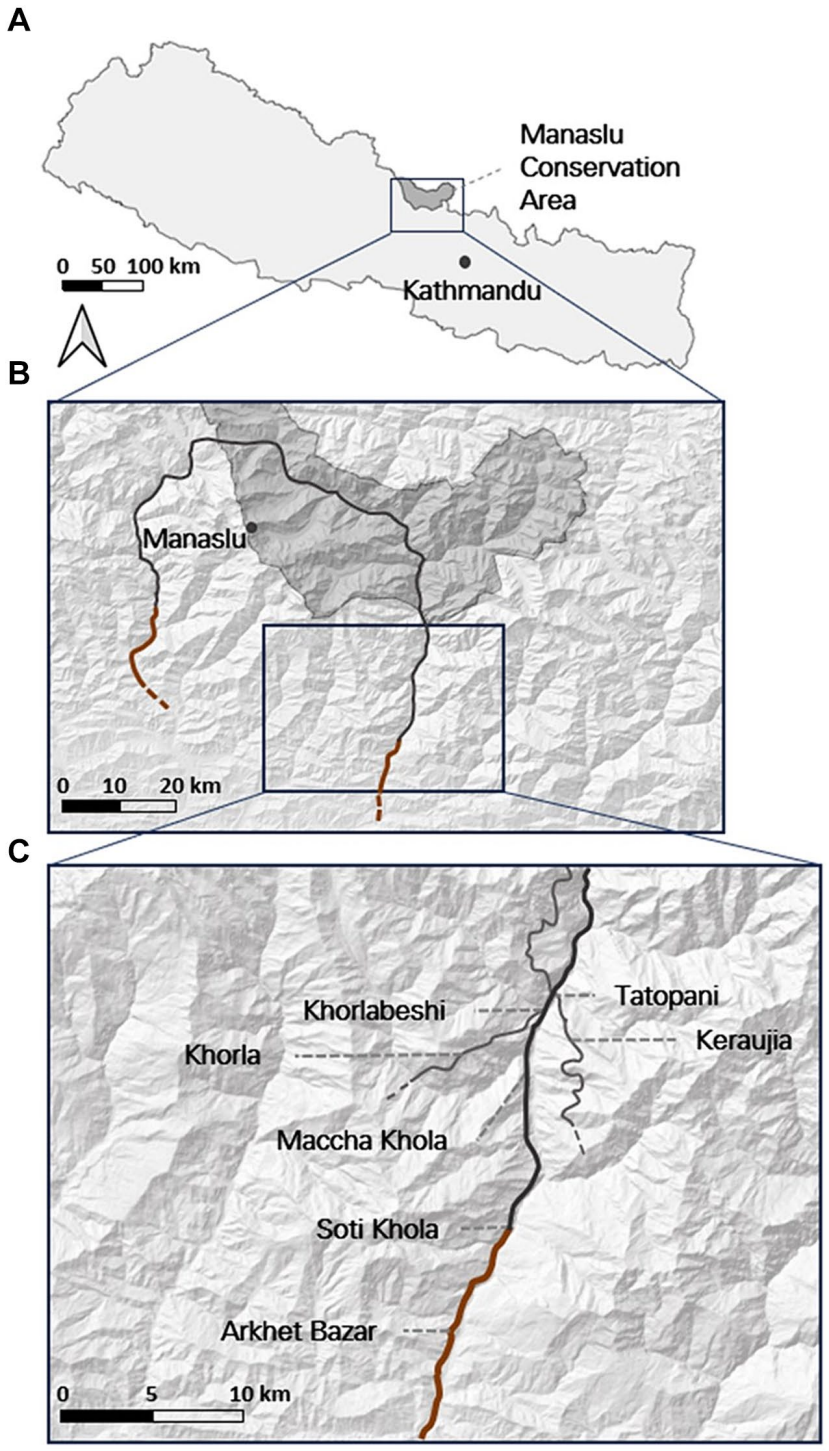
Location of study sites visited during 2018 to assess the role of mountain mules and the livelihoods of local residents. Sites are located within **(A)** the Manaslu Conservation Area, Nepal, **(B)** along the first part of the Manaslu Circuit Trail, **(C)** with specific locations either on the main trail or along alternate paths along the trail. Paths are accessible by vehicle (orange) or by foot or mule only (grey).

Fieldwork was conducted from 12th to 25th November 2018 in Gorkha, Nepal. Study sites consisted of several communities along the main Manaslu Circuit hiking trail, a popular trekking route for tourists. A number of guesthouses are found in each of the villages along the route, providing food and accommodation for tourists and locals, as well as the mule owners and drivers that travel along the trail from Arkhet, providing supplies. Mules have been commonplace in the Manaslu mountain area for approximately 12 years. They transport goods along the entire trail from Soti Khola, after which the tracks are no longer accessible by motorised vehicles, as well as to the smaller villages dotted along the steep valley sides. Communities included in the study were Arkhet Bazar, Soti Khola, Machha Khola, Khorlabeshi, and Tatopani, and two sites a short distance from the trail, Khorla and Kerauja (Figure 1). Arkhet Bazar and Soti Khola were accessed by vehicle, while the remaining study sites were only accessible on foot or hoof.

### 2.2 Participant outline

Participants included mule owners and ‘drivers’ (who are employed by mule owners to work with their mules), as well non mule-owning members of each community. Interviews were conducted with non mule-owners to understand the indirect role of mules in supporting their lives or livelihoods. As the human population throughout the valley is very low, participants were recruited based on their availability, and stratified by mule ownership and non-ownership in each community wherever people from both groups were present. In Arkhet Bazar, mule owners and drivers were approached while attending a veterinary intervention clinic organised by Animal Nepal, whilst those in other locations were sought on an *ad hoc* basis. The veterinary clinics are a regular service provided by Animal Nepal, where mules are checked and treated for any health issues. Although sourcing mules from this clinic may have introduced a selection bias for mules with existing health issues, or owners who were particularly concerned about their mules welfare, we feel that this bias is minimised by the fact that they take place in the main mule resting area in Arkhet Bazar, and few mules were observed outside of this area in this particular location.

### 2.3 Data collection

A standardised livelihood survey gathered information on demographics, employment and income, details about working practises, and handling experience of the owner. Each survey was recorded electronically on a digital device using an Open Data Kit (ODK) Collect form (18) containing pre-set questions (see Supplementary material).

Semi-structured interviews (SSIs) were collected immediately following the livelihood surveys and explored four broad topics of; people’s background and history of the area; the socio-economic role of mules; and sustaining livelihoods. While interviews were designed around these categories, their semi-structured nature allowed them to develop according to the responses and interests of participants. For example, as the impacts of the monsoon and new road construction became prominent features of initial interviews, these were added to subsequent interviews for further exploration. Interviews lasted from 20 to 54 min, with the length of each interview determined by the availability of the participant. SSIs were conducted by principal researchers in English and interpreted to and from Nepalese *in situ* by Nepalese interpreters. Interviews were recorded and the English interpretation was later transcribed. All interviews were conducted in a location of the participant’s choice with a single interviewee, apart from one group of four mule owners/drivers who were interviewed together.

To assess the links between owner handling experience and mule welfare, owner experience data were collected during livelihood surveys as the number of years of experience working with mules (‘experience’), whether or not the owner had worked as a driver prior to owning their mules (‘prior knowledge’), and whether the mules were currently being worked by a driver or the owner themselves (‘current handling’). The latter two cofactors were included to assess whether the owner had received guidance from an existing mule owner, and to take into account the current experience of the mule, respectively. Mule behaviour was recorded using the question ‘Presence of signs of fear and distress’ during EARS assessments [Equid Assessment Research and Scoping; (19)], where signs of fear and distress were recorded when one or more of the standard behaviours outlined in the EARS protocol were observed (see Supplementary material). EARS is a welfare assessment tool covering physical and behavioural aspects of welfare and was conducted concurrently with surveys and SSIs as part of a wider study [see (11, 20)]. All mules were assessed while at rest. The mule was first observed for any signs of fear and distress from a distance, but any signs that occurred during the entire assessment, including while the observer approached the mule, were recorded. This assessment approach is standardised as part of the EARS assessment and was conducted consistently for all mules. Assessments were conducted by a trained welfare assessor. Due to the nature of the study location, it was not possible to complete assessments for the mules of all owners as some mules were not present at the time of interview or were removed to start work before assessments could be completed.

### 2.4 Data analysis

SSIs were analysed following Braun and Clarke’s six-step process of thematic analysis (21), where initial codes were created using a deductive approach using these pre-determined research topics to ensure focus on the main research questions (22). Inductive coding was also conducted to allow further themes to emerge from the data. Coding into both node-types was conducted simultaneously and as new themes emerged. Coding was performed in several iterations, and new themes and sub-themes were noted wherever they appeared (23). The analysis was repeated until the coding was considered complete and no new themes emerged. Inductive themes of ‘opportunity and diversification’; ‘reliance and risk’; and ‘transience and the future’ were determined. All semi-structured interview transcripts were analysed using thematic analysis in N-Vivo (V12.2, QSR International). A second researcher was consulted at intervals to review emerging themes and codes.

The effects of owner experience on mule behaviour were analysed using a generalised linear mixed effects model (GLMM) with binomial (logit) distribution. Responses to the EARS assessment question ‘Presence of signs of fear and distress’ were re-coded to ‘fear present’ or ‘no signs of fear’ where appropriate. ‘Prior knowledge’ and ‘current handling’ were included as fixed factors, and ‘experience’ was included as a fixed covariate. Owner ID was included as a random factor. An interaction term between ‘prior knowledge’ and ‘experience’ was included to fully explore the effect of the owners experience, but was found to be non-significant and removed. For all analyses, we present estimates of the full model to avoid bias associated with stepwise deletion of non-significant terms (24). We present likelihood ratio test results for the deletion of each term from the full model (25, 26). Prediction uncertainty of the full models is calculated using *N* = 1,000 random draws from the estimated parameter distributions and presented as the 95% quantiles of the resulting distributions (26, 27). Analyses were performed in R version 4.3.1 (28) using R Studio (29).

### 2.5 Ethical considerations

Participation was voluntary and unpaid, and all participants were over 18 years old. Participants were not recruited in advance of the field period. To ensure inclusion of participants regardless of their level of literacy, consent was obtained verbally and audio-recorded. Respondents were provided with a code to enable researchers to link survey and interview data, whilst retaining participant anonymity. Participants were provided with contact information for Animal Nepal and the right to withdraw within 2 weeks of data collection.

The study and protocols were conducted in accordance with the Declaration of Helsinki (30) and were approved by the Research and Ethics Review Committee (RERC) of The Donkey Sanctuary, UK; project Number 2019-AIM2-NEPAL.

## 3 Results

### 3.1 Descriptive statistics

Semi-structured interviews were completed with 55 participants, 27 of whom owned or worked with mules (where two mule owners and two mule drivers who were interviewed together as a group).

Livelihoods surveys were completed with 23 mule owners and 26 non mule-owning participants; four participants who worked with mules (the group interviewed together) and two non-mule owners did not complete surveys due to time constraints where participants had other commitments. For these participants, details about mule ownership and job role were extracted from SSIs.

Non-mule owning participants had varied job roles, with six participants holding more than one job role. Six mule owners held alternative roles as shop or teahouse owners; four of these participants listed their other (non-mule) business as their main source of income (Table 1).

**Table 1:** Job roles of non-mule owners and mule owners (excluding mule work) in the Manaslu Valley, Nepal.

	Non mule owners		Mule owners	
	*n*	%	*n*	%
Vet tech	2	8		
Community leader	1	4		
Teahouse owner	6	23	2	9
Shop owner	7	27	4	17
Farmer	7	27		
Tourist guide	1	4		
Teacher	4	15		
Social worker	3	12		
Government worker	1	4		
Student	1	4		
Construction worker	1	4		

Percentages are for the number of people fulfilling a job role out of the cohort of either non mule owners (*n* = 26) or mule owners (*n* = 23) for which livelihood data were available. In some cases, participants held more than one job role, so percentages may not total 100.

Of the participants that completed livelihoods surveys, 22% of mule owners (*n* = 5), and 62% of non-mule owners (*n* = 16) were female. The majority of mule owners were aged 31–50 (*n* = 15; 65%), with seven mule owners aged 18–30 (30%) and one aged over 50 (5%). Non mule owners were mostly 18–30 (*n* = 12; 46%) or 31–50 years old (*n* = 11; 42%), with three non-mule owners being over 50 years old. Mule owners reported to owning a mean of 14 mules each (SD = 7).

### 3.2 Background to life in the Manaslu Valley

One respondent, a shop owner who had lived in Arkhet for 24 years and had helped create the tracks that originally led through the village, outlined life before the presence of working mules. People relied solely on hired porters, at great expense, or on themselves to carry supplies such as petrol, food, clothes, and other necessities. People would regularly walk as far as Kathmandu for a supply run, where one person would carry between 40 and 60 kg over 2–4 days for the round trip of approximately 130 km. Agriculture was more prominent in the mountains then, with farmers completing the journey to Kathmandu on foot to sell rice. Infrastructure in these remote mountain communities was limited, with people lighting corn cobs for light and growing crops or raising animals for food more frequently than today, supplementing with foraged foods and herbal medicines from the jungle.

Whilst agriculture and livestock farming currently occur within the valley, several respondents reported that the steep terrain limits the amount of land available without terracing and frequent landslides, particularly during the monsoon season, which limits their output. One family reported that their entire farmland had been wiped out the previous year in a landslide that killed one member of their family, and they no longer grew their own crops.

### 3.3 The socio-economic contribution of mules

Guesthouse and shop owners throughout the valley reported that they rely on mules to transport their supplies and stock, and locals order food and necessities in bulk, thereby supporting SDG 2.1 ‘Zero Hunger’ (Table 2). Mules also supply gas cannisters, stationery and reading material for the local schools, construction materials (SDG 9a ‘Industry, Innovation and Infrastructure’; Table 2), and some medical equipment, although these tend to be carried by porters to avoid breakages. A small number of farmers use mules to transport crops from farm to market, although the majority of the cohort only grew food to eat themselves, as fodder to sell to mule owners, or to use to make a local wine.

**Table 2:** The United Nations Agenda 2030 Sustainable Development Goals supported by the presence of mules in the Manaslu Valley, Nepal.

SDG		Evidence for support
1.4	No poverty	Most mule owners felt that mule work was their ‘only option’ in communities where options for income were very limited
2.1	Zero hunger	Mules transport food and supplies for more affordable rates than was previously possible through employing porters at greater expense
4.1	Quality education	Supplies brought by mules enabled a local school to stay open for an extra 2 months per year
8.6	Decent work	Mules provide an income where options are limited, including for respondents who had previously sought work overseas, and free up time for community members to build other businesses where time and money was previously consumed by transporting essential supplies
9a	Industry, innovation and infrastructure	Materials to build earthquake resistant housing are transported via mule to remote communities throughout the mountains
11.5.3	Sustainable cities and communities	Whilst the risks must be managed, mules provide an opportunity for income during the monsoon season when the price of goods is inflated, and reduced disruption to services for residents who would otherwise be relatively cut off from support

Mule owners and drivers tend to work with approximately 10 mules at a time, and people requiring goods must hire all of the mules, known as a ‘mule train’, at once. Local people often club together to order enough supplies to make the hire of a mule train worthwhile, although it wasn’t uncommon for respondents to hire an entire train themselves to stock up on 6 months’ worth of staples such as rice and salt. Sixty-four percent (*n* = 16) of mule owners reported that they worked with the mules themselves, while 36% (*n* = 9) hired a mule driver to work and tend to the mules on the owner’s behalf.

Mule owners reported that transport of goods costed approximately one rupee per kilogram per kilometer from Maccha Khola, where goods are deposited by vehicle and stored. Costs are increased during the monsoon season depending on the level of stock, the weather, and the conditions of the tracks. Stock is managed and costs are decided by the equine community leaders, while individual jobs are organised by the equine owners themselves. The current equine community leaders, two brother-in-laws based in different villages on the main trail, have been in the roles for the last 7 years. These two leaders were voted into the roles by the equine owners, who attend regular meetings, and were expected to stay in the roles until someone else comes forward. According to one equine community leader:

“*We are the main community leaders so we work together. Everybody is pleased for me to be the [community leader], so I cannot say no, so I am happy. I think it should be changed so everybody gets the chance to be community leader. In the meetings, whenever [they are] held, we keep on telling people ‘you should be the one’, but if nobody comes up, then I will be here*.”

While the vast majority of respondents spoke positively about the role of mules, one non mule-owning respondent did not feel the mules had made a positive contribution to life in the valley, as they had increased access to fast food, and decreased the incentive for people to grow their own food. Other respondents, however, felt that the mules were not the main deterrent to growing crops, given the uncertainty and difficulty associated with successfully cultivating the land in the area, and noted that the increased access to a wide variety of food was a positive contribution.

### 3.4 Sustaining livelihoods

When asked about why they had originally started working with mules to transport supplies, many mule owners felt it was the option that allowed them to provide for their family, supporting SDG 1.4 ‘No Poverty’ (Table 2):

*“I was responsible to make my family’s financial position strong, and here in the village I couldn’t get any other job and so I [was] compelled to choose this one*.”

“*I cannot carry the loads by myself, so I decided to work as an equine owner so that we can have a business here*”

During the early years of mule presence in the mountains, people began to choose mule trains to transport their goods over the services of porters. As a result, several former porters within the cohort transitioned into working with the mules. For 24% of mule owners (*n* = 6), this progression included an intermediary step of becoming a mule driver, whereby respondents learned the skills needed to care for their own mules from an existing owner. While this transition was planned in some cases, two of these former drivers decided to buy mules after realising that they could earn more money if they bought their own. According to one mule owner, who had previously worked as a mule driver:

*“As I had been working for two years with my mule’s owner I had a very low basis salary, so I have an idea that as soon as I knew how to handle it, then I’d start my own [business], even though I need to take a loan from others.”*

Three respondents had previously travelled abroad (e.g., to Saudi Arabia) to carry out construction work, as they felt this would be more lucrative than any work they could find at home. All of these respondents (one mule owner and two drivers) returned home once they discovered, through friends or acquaintances working with mules, that they could make more money at home by working with mules as well.

One respondent indicated that at least 12 mule owners living near their village, which is located off the main track had initially purchased mules as a response to increased demand after the 2015 earthquake.

“*Previously [immediately after the earthquake] there wasn’t enough mules, but later on when the tracks were fine there were three or four hundred mules added*”

The respondent added that demand for construction materials was ongoing as houses were still being repaired and rebuilt, but they were unsure as to the fate of these mules once repairs were completed. As reported by one of the equine community leaders, people in other parts of the valleys also responded to demand by purchasing mules, although it is not clear how many of these people were new to the trade.

The majority of mule owners were satisfied with their work, stating that they earned more than in their previous professions as porters, dishwashers, farmers, working abroad on construction sites, shopkeepers, assistant tourist guides and, in one case, as a police officer. Working with mules was considered to be easier than working as a porter. Mule owners reported that the work provides more freedom than being employed by other people.

Whilst none of the other mule owners expressed regret at their decision to purchase or work with mules, several discussed the risks involved with this type of work. Many had lost mules as a result of falls down the steep valley sides, or feared this possibility. One owner expressed that, whilst they did not regret their choice to purchase mules (due to its necessity), they would rather find another opportunity for work. They felt that the job was too risky for both people and mules, with the high chance of landslides or falls from the steep, often unstable, tracks; as well as the potential loss of mules due to illness. This respondent, however, felt that they had no other options for work that would provide the income they needed, and so did not have plans to change profession:

“*[I purchased mules] for my children’s schooling and education. I need to have some money so I need to do this profession. My children’s schooling is here so I need to stay here. Previously I worked as a labourer. Basically, I like my previous profession because this profession [mule work] seems very risky, and the route is very dangerous*”

### 3.5 Experience and mule welfare

Sixteen percent (*n* = 4) of mule owners bought mules as a result of a personal connection, where either an acquaintance or friend who already had mules introduced the respondent to the work. Only one respondent said that a family member had introduced them to mule work when they became a mule driver for their brother-in-law. None of the respondents had grown up in a family that owned mules or had the skills passed down to them from their immediate family.

Data from 14 mule owners and 127 mules were available to assess the effect of level of experience, prior handling experience and current handling on levels of fear and distress in mules (Table 3). The prevalence of signs of fear and distress was not significantly different between mules owned by participants who had previously worked as drivers (60% of mules, *n* = 21) and those who had not worked as drivers (54%, *n* = 50, Table 3). There was, however, a marginally significant difference in the prevalence of fear and distress depending on who currently worked with the mules; those worked by drivers were less likely to show signs of fear and distress (42%, *n* = 16) than those worked by their owners (62%, *n* = 55, Table 3). Signs of fear and distress were also more prevalent in mules that had more experienced owners (Table 3); owners that had worked with mules for 9 years owned the majority of mules that presented signs of fear and distress, while the majority of mules that did not show signed of fear and distress were owned by those who had worked with mules for 4 years. For the subset of owners included in the model, experience of working with mules ranged from 1 to 18 years (mean = 7.9 years, *n* = 14).

**Table 3:** Coefficient estimates for the GLMM to assess signs of fear and distress in mules in the Manaslu Valley, Nepal.

Predictor	Estimate ± SE	*χ* ^2^_df_	*p*
Intercept	0.27 ± 0.51		
Current handling (handler)	1.44 ± 0.65	4.39_1_	**0.04**
Prior knowledge (yes)	0.12 ± 0.65	0.04_1_	0.85
Experience	−0.17 ± 0.06	7.86_1_	**<0.01**

Estimates are for the full model. Log-likelihood *χ*^2^ statistic and associated *p* values are for the deletion of each term from the full model. *p* values for terms significant at the <0.05 level are presented in bold.

### 3.6 Opportunity and diversification

Opportunities for ‘decent work’ (SDG 8.6; Table 2) were reported by several respondents as an indirect result of the mules presence. Two teahouse owners had started their businesses in response to demand by mule owners for a place to stay. One of these respondents knew many of the mule owners from their time working as a mule driver:

*“[People] tell me to do the other things like the people used to do, like labour, [but] I cannot do that because I have a small kid. The mule drivers understand my language, they used to come here to stay, and slowly other people started to come there with them. I cook food for them, give them dinner.”*

The time gained from being able to transport supplies by mule, as opposed to carrying supplies themselves or hiring porters, has afforded several non mule-owners with an opportunity for diversification. For example, one teahouse owner had begun to grow their own food as they no longer had to take a day per week to transport supplies themselves, which they had opted to do as porters were deemed too expensive.

Three teachers from the school in Khorla described mules as a critical component in keeping the school running (SDG 4.1 ‘Quality Education’; Table 2). The school hired a mule train that brought supplies, including books, stationary and teaching materials to the school five to six times per year. These deliveries enabled the school to retain enough teaching supplies, and for teachers to reclaim enough time, to stay open for an extra 2 months per year during the monsoon, when they previously had to close.

### 3.7 Reliance and risk

The impact of the death of a mule was felt emotionally as well as financially, with owners recounting ‘feeling lost’ after the loss of a ‘beloved mule’, and that they needed to take out loans or use long-term savings to replace the mules. One owner said that the loss of a mule had led to heavy drinking. The risks involved with the job undoubtedly caused a great deal of stress for many participants. Some respondents felt that, should they lose their mules unexpectedly due to illness or accident, they would need to sell their home and move somewhere with more opportunities for work. Others had not considered moving for work, stating that ‘my house is here’ and they would do whatever was needed in order to stay.

For a minority of mule owners, the financial impacts of losing a mule were mitigated by other sources of income. Twenty eight percent (*n* = 7) of mule owners also owned a shop or guesthouse (Table 1), which they stocked using their own mules, but could keep running by hiring other mule trains if they experienced an unexpected loss. Few mule owners worked with their mules during the monsoon, reporting the risks as being too high and the unpredictable nature of the monsoon difficult to work around. These owners reported managing their earnings from the rest of the year to last through the monsoon season, although several reported having a ‘financial crisis’ and finding it difficult to manage without an income during the monsoon season. These difficulties are mitigated somewhat by an increase in demand prior to the monsoon season, when locals and business owners stock up on staples to avoid the higher prices of transport during the season.

A minority of owners continued to transport supplies during the monsoon, either through necessity as their only source of income…

*“It’s so hard to work during the monsoon, [but] if I stopped for the whole monsoons saying that it’s raining, and [during this period my] mules are eating, I am eating and I am not earning anything”*

…or as an opportunity for higher pay while fewer mule owners were working – the price per kilo can be almost doubled during this time. As well as the parts of the trail normally accessed by mule, during the monsoon mules are required to transport supplies along the initial parts of the trail that are usually accessed by vehicle, as the road becomes extremely difficult to navigate in a motorised vehicle after heavy rain. Whilst the risks associated with working during the monsoon must be managed, mule transport does provide the potential for a relatively sustainable income for some mule owners, and the continuation of a basic supply service to the area which non-mule owners reported as being useful in times of need. This service provides tentative support for SDG 11.5.3 ‘Sustainable Cities and Communities’ by reducing the disruption to basic services often caused by extreme events (Table 2).

The majority of tracks, particularly those off the main tourist trail, are relatively unstable and easily damaged by heavy, persistent rain during monsoon season. Mules have been reported to cause damage to the tracks during this time, although this damage is likely to be minimal compared to the landslides caused directly by the extreme weather.

### 3.8 Transience and the future

At the time of fieldwork, the road network planned for the Manaslu Valley had been completed as far as Soti Khola (Figure 1) and is likely to be completed through the rest of the valley in the next few years. Arkhet Bazar used to mark the end of the road passable by motorised vehicles. It acted as the main hub from which the Manaslu Circult Trek would begin, and stocks of supplies would be kept ready to be transported through the Manaslu Valley by mules. Tourists, as well as supplies, are now able to travel to Soti Khola via vehicle, and the role of mules has been displaced to locations further along the trail.

The construction of the road through Arkhet Bazar has had a series of impacts on livelihoods in the area. We found very few teahouses in Arkhet Bazar during fieldwork. One of the few remaining teahouse owners reported that, as well as catering for tourists before the start of their trek, mule owners would stay with them, and other teahouses in the village, between supply runs. Following the road construction, neither tourists nor mule owners needed to stay there and instead travelled directly to Soti Khola. Whilst the tearoom was still open, the respondent did not feel that it could stay that way for much longer, though they had not planned what to do next. Another teahouse owner, who solely catered for mule drivers, was in a similar situation:

*“Previously there used to be a tent for mule drivers [to stay] every day and more mules, but now the number of mules is decreasing as well as the number of people coming to my place. Only one or two mule drivers come here, and they order the food, that’s it. I can’t leave [my family] hungry. I don’t think the mule people will come in the future, so I’ll do whatever I can do, I’ll do something else […] until now I haven’t thought of anything.”*

When asked about their plans for the future, while acknowledging that the road was likely to affect them, the majority of mule owners in locations further along the trail had not decided what they would do to secure their livelihoods. The few that had made plans either wanted to open a teahouse or shop, or buy livestock to farm. One respondent had already bought a shop, using earnings from work with their mules, in anticipation for reduced demand for mule work. Another respondent wanted to continue to work with the mules until their debts were paid, then train to become a tourist guide.

Most mule owners, however, were not concerned with the road construction as they felt there would be enough remaining demand for mules to travel to villages perched higher up the steep valley sides off the main tourist route and across into neighbouring valleys. These routes have the potential of becoming more popular with tourists as tour guides begin to offer new ways for people to enjoy the mountains away from the road. However, as the equine community leader based in Soti Khola reported:

“*People are thinking about making alternative routes, so we can at least transport to those villages, but later on, slowly, we know there will be a road in those villages too. I used to live in a different village but a road [was built] through, so I came here. I don’t know, I might go further up the track or do something else*”.

The equine community leader based in Maccha Khola was slightly more optimistic in terms of the potential need for mule work, albeit with increased risk to both humans and mules:

*“Even in the future there will still be mules working to carry the firewood and stones from the jungle, as previous. After Soti Khola it’s like the place is cursed with the landslides. There will landslides for sure, so they’ll always need the mules*.*”*

Mule owners acknowledged that they would likely have to accept a higher level of risk following the road construction if they were to continue working in the area, with some stating that they would start working during the monsoon.

For residents of Tatopani, one of the main stops for trekkers enroute through the valley due to the hot springs, the road is highly likely to cause widespread displacement and disruption. According to SSI respondents, several houses, along with the hot springs, will likely be irreparably damaged or destroyed to accommodate the road. One shop keeper, who currently employs one full mule train per week, sells an equal amount of goods to locals and tourists. When asked how she would be impacted by the availability of vehicles to transport goods, she reported:

*“It doesn’t matter if the supplies are cheaper, if the people are unemployed. After the road is built, only a few people [will] stay here, most of them will go up in the vehicles. Even the locals will not stay here.”*

## 4 Discussion

### 4.1 Sustaining livelihoods

The movement of goods via pack animals is one of the oldest forms of transport in the world (4), and has supported the development of permanent communities in areas in which it would otherwise be difficult to survive. In the Gorkha region of Nepal, mules support an entire community infrastructure, from supplying families with food and essential items (SDG 2.1), stocking businesses such as guest houses in order to sustain the tourism industry (on which many residents rely) to supporting the livelihoods of the mule owners themselves where there are few options for sustainable work (SDG 1.4). The transition from hiring porters to employing mules and their owners to transport goods has granted both money and time to non mule-owning respondents, many of whom would carry these goods themselves due to the high cost of porters. Several respondents dedicated this time to diversifying their income by starting other businesses (SDG 8.6) or supporting local services such as enabling the school to open throughout the year (SDG 4.1; see Table 2).

Many respondents purchased mules in response to increased demand, such as to aid recovery from the 2015 earthquake. Working mules provided people with the ability to adapt and generate an income while supporting the community in long term recovery (8). However, when mules are ‘seen’ purely as a source of income they can become invisible; for example, where mules have a role in tourism, welfare issues have been exacerbated as their role is controlled externally *“the mule, in some circumstances, can be ‘controlled’ by outsiders who may see the mule differently from the mule owner and/or handler. The mule risks becoming an object for exploitation as a pure means to make profit in the short term*” (31). Although there is some recognition of the role of working equids in sustainable development, the intrinsic links between equid and human health and wellbeing and human development within a ‘One Welfare’ framework (32) are currently poorly recognised. The recent proposal of an 18th SDG on animal health, welfare and rights, provides a promising move towards integrative development (33), where animal concerns are valued alongside human ones.

### 4.2 Experience and mule welfare

Perhaps surprisingly, both owners with more experience of working with equids, and those who worked with their mules (as opposed to hiring drivers), were more likely to own mules with poor behavioural indicators of welfare, and prior handling experience did not result in healthier mule behaviour. According to respondents, mules are relatively new to the Manaslu Valley, having been introduced around 12 years ago. Historically, mule trains, and the social networks around them, were considered part of the cultural institution of some parts of the Himalaya. Local teahouses provided food and lodgings for both humans and their equid counterparts (34, 35). With the development of road networks, this socio-cultural practise has all but degraded, leaving isolated pockets of support for those travelling by foot or hoof. A loss of traditional knowledge transfer systems may, therefore, limit the in-depth experience new mule owners can gain from their peers (36). While mule owners in the Manaslu Valley had, in some cases, been working with their mules for over a decade, the lack of ingrained cultural or social norms around animal care, coupled with a lack of veterinary support in the area [see (37)] may mean that even relatively experienced owners have no basis of knowledge or support on which to draw. Potentially, those who have worked with mules for longer may have also developed poor handling techniques in the absence of peer support, or become fearful of the mules they are handling resulting in more aversive handling techniques which, over time, could lead to defensive mule behaviour (20, 38). External education programmes could be of value here, although these must be targeted at encouraging knowledge sharing through local networks to avoid ‘knowledge fade’ once the programme is complete (39). To do this, delivery methods must be tailored to the local demographic, taking into account the unique cultural and social structures within each community and ultimately aim to embed ownership with key actors within the community itself (37, 40).

Several other factors have been found to impact human-equid interactions, including human perception and empathy, owner locus of control, and levels of poverty (37, 41, 42), so it is likely that other factors that we did not account for had an impact on mule behaviour. Prior to working in the mountains, mules in the current study area predominantly worked in brick kilns in India (20). Despite the working conditions in the kilns being very different, many owners considered the mules to be trained and provided very little time for habituation to the new working regime, which may adversely affect their behaviour. When training was provided it was somewhat aversive, with reports of ‘dragging’ mules across unfamiliar suspension bridges, which may have resulted in a poor human-equid relationship in some cases (20).

### 4.3 Reliance and risk

Concern about the large-scale but relatively distant changes from the road construction did not feature as prominently as concerns regarding the more immediate risks associated with the monsoon, and the dangers of walking on unstable tracks as part of a working day. As well as the inherent physical dangers associated with mule work to both mule and owner, the immediate consequences of mule loss were substantial for both the financial security and mental wellbeing of mule owners, with inevitable links between the two (43). Several respondents felt they had ‘no other option’ for work, which could have both positive and negative consequences. Whilst people have access to a sustainable way to make a living, it may also result in mule owners and drivers accepting a higher level of risk than they may feel is acceptable should alternate work be more readily available.

Marginalised communities often face a greater exposure to livelihood threats than affluent ones (44). People living in the margins, particularly in rural or remote locations, tend to have less of a financial buffer to absorb any unexpected loss of income, are less likely to belong to a social network that could offer support and often lack the resources to mitigate the impacts of harsh environmental conditions or ‘shock’ events (45). As such, income diversification is often crucial to these communities, as people seek to absorb some of the financial risks associated with seasonal or uncertain work whenever they are able to find alternative sources of income (46). The presence of mules in Manaslu Valley has enabled participants to diversify their income in several cases within our cohort. Some mule owners, however, were more likely to take higher risks within their current profession, such as choosing to work during the monsoon where they have the opportunity to earn a premium, to mitigate the impact of uncertainty or change. Unfortunately, the current risks associated with mule work are likely to increase with the construction of the road. Landslides occur more frequently along new road corridors (47), increasing the risk of fatalities and potentially disrupting supply chains, and livelihoods, from mule transport as well as vehicles. These risks create a vicious cycle whereby mule owners are constantly balancing a reliance on income from mules and the evolving risks and uncertainties involved with the work itself.

### 4.4 Transience and the future

Road developments can be life changing for remote communities. Changes are often dynamic, with impacts in environmental, sociocultural and socioeconomic spheres shifting through time (48). The Mustang district of Nepal, for example, experienced a severe decline in the number of mules, the employment of their owners in the trade, as well as in the prevalence of traditional enterprises catering to their needs following road construction throughout the district (49).

Whilst a minority of mule owners in the current cohort either had a secondary source of income, or planned to start a new business (e.g., teahouses), most were solely reliant on the income from mule work and had not decided what they would do once the road was built. Some participants, such as those living in Tatopani, may have no option but to move elsewhere. This shift may signal a loss of connection or sense of belonging, particularly for the younger generation, which could have disastrous effects for the conservation of the fragile and diverse ecosystems found in the Manaslu Conservation Area where local people act as custodians. As Shant Raj Janwali of the U.S. AID-supported WWF-Hariyo Ban “Green Forest” Program states in Coburn (15), “*The conservation value of one’s personal attachment to their homeland cannot be overstated, and this goes missing as people move away*.”

In the lower section of the Manaslu Circult Trail, roads have replaced the tracks which used to function as both tourist trekking routes and tracks for mule transport. Tourists now bypassed villages such as Arkhet Bazar, which used to serve as tourist hubs, in favour of villages further along the trail where the road ends and hiking trail resumes. This change has resulted in a loss of income for businesses that supported both tourism and the mule network itself. At the time of writing, the road in the current study area had been completed as far as Khorlabeshi, and was passable to Maccha Khola during the monsoon (Animal Nepal, pers. comms). The impacts seen in the current study area mirror those found along the Annapurna Circuit Trail (ACT), where roads have now replaced the majority of what was once the main trekking route (50). Tourists numbers reportedly reduced in Annapurna following the road construction, as travel agencies directed people to other destinations which were not affected by road construction (49). Over time, however, much of the ACT was rerouted, with several alternative hiking options near to the original trail provided by the Annapurna Conservation Area Project (National Trust for Nature Conservation). This recovery reflects several mule owners assertions that demand would still exist elsewhere in the valley after the completion of the road. Indeed, some trekking agencies have already started offering alternative hiking routes very close to the original Manaslu Circuit Trail (51).

## 5 Conclusion

While the future remains uncertain, the people living in the Manaslu Valley, Nepal, demonstrate resilience in the face of extreme environmental and social challenges, made possible through the partnership they share with their mules. Mule work has enabled people to find a source of income where they felt they had no other option within their community; diversify their income to better absorb potential financial strain; strengthen community resources such as the school; and mitigate the impact of disaster through the use, and expansion, of existing mule transport networks.

According to the United Nations Development Programme, current definitions of resilience need to expand their focus from response to prevention and preparedness (52). This expansion is particularly important for mule owners in the Manaslu Valley where, in the face of financial hardship, mule owners may opt to replace financial risk with physical risk to both themselves and their mules. Humanitarian aid programmes with a focus on mitigating the impacts of crises and preparing for change align with SDG 1.5, which aims to build resilience by ‘reducing exposure and vulnerability’ to extreme events and to social and environmental shocks (8).

When animals play a central role in recovery or resilience in times of disaster or uncertainty, the animals themselves often become invisible; their welfare often takes a backseat and they become a commodity, particularly where their use is expanded to people who do not have access to the support networks or generational knowledge required to maintain equids in a good state of welfare. We support the formalisation of an 18th SDG on animal health, welfare and rights (33), and argue that a focus on improving and maintaining mule welfare, through the expansion of knowledge sharing and support networks within and between communities should form an integral part of actions aimed at supporting communities to prepare for expected and unexpected change.

## Data availability statement

The data presented in the study are deposited in the UK Data Service ReShare repository, DOI: 10.5255/UKDA-SN-857303.

## Ethics statement

The studies involving humans were approved by The Donkey Sanctuary Research and Ethics Review Committee (RERC). The studies were conducted in accordance with the local legislation and institutional requirements. Written informed consent for participation was not required from the participants or the participants’ legal guardians/next of kin because informed consent was obtained verbally and audio recorded to allow access for participants with different levels of literacy. The animal studies were approved by The Donkey Sanctuary Research and Ethics Review Committee (RERC). The studies were conducted in accordance with the local legislation and institutional requirements. Written informed consent was not obtained from the owners for the participation of their animals in this study because Informed consent was obtained verbally and audio recorded to allow access for participants with different levels of literacy.

## Author contributions

LK: Conceptualization, Data curation, Formal analysis, Investigation, Methodology, Project administration, Resources, Validation, Visualization, Writing – original draft, Writing – review & editing. TW: Conceptualization, Data curation, Investigation, Methodology, Project administration, Resources, Validation, Writing – review & editing. ST: Data curation, Investigation, Methodology, Project administration, Resources, Writing – review & editing. CN: Formal analysis, Resources, Validation, Writing – review & editing. NC: Conceptualization, Methodology, Writing – review & editing.
