# Supplementary material for: Mule trains to mountain roads: the role of working mules in supporting resilient communities in the Himalayas
Source: Front Vet Sci. 2024 Jul 31;11:1390644. doi: 10.3389/fvets.2024.1390644 (PMC11322082; doi:10.3389/fvets.2024.1390644)
Supplement: Supplementary file 1 [file Table_1.docx]

Supplementary Material

Mule trains to mountain roads: the role of working mules in supporting resilient communities in the Himalayas

**Kubasiewicz, L.M.*, Watson, T., Thapa, S., Nye, C, Chamberlain, N.**

*** Correspondence:** Laura Kubasiewicz: laura.kubasiewicz@thedonkeysanctuary.org.uk

# EARS guidelines: Fear and Distress

Guidelines for the question “Please indicate signs of fear and distress present” in the Equid Assessment, Research and Scoping (EARS) welfare assessment tool. See Raw, Rodrigues et al. (2020) for full details of the EARS tool.

## CATEGORY 4F - Fear and distress

| QUESTION 4-29 | Please indicate signs of fear and distress present (MC) |  | 1 - No signs of fear and distress present 2 - Showing the whites of the eyes  3 - Unpredictable or sudden movements  4 - Sudden ‘startle’ responses when standing quietly (rapidly lifting the head) 5 - Aggressive behaviour  6 - Trembling 7 - Head shyness 8 - Completely withdrawn/shut down  9 - Other (please specify) |
| --- | --- | --- | --- |

NOTES
The equid displays obvious signs of fear/distress during the time it is being observed. Multiple options may be selected.

Question Choices

| QUESTION 4-29 | 1 - No signs of fear and distress present |  |
| --- | --- | --- |
| QUESTION 4-29 | 2 - Showing the whites of the eyes | The sclera of the eye is easily observed. |
| QUESTION 4-29 | 3 - Unpredictable or sudden movements | The equid makes sudden movements or reacts to external stimuli (such as the observer, other animals or objects within the environment) with rapid, sudden movements or avoidance behaviours. |
| QUESTION 4-29 | 4 - Sudden ‘startle’ responses when standing quietly | The equid is standing quietly but intermittently ‘startles’ – this is generally observed as the equid suddenly and quickly jerking the head upwards. |
| QUESTION 4-29 | 5 - Aggressive behaviour | The equid demonstrates aggressive behaviour towards the observer, other animals or objects within the environment. This may be demonstrated through bite/kick threats or actually biting or kicking out. |
| QUESTION 4-29 | 6 - Trembling | The equid’s body can be seen trembling or shaking. |
| QUESTION 4-29 | 7 - Head shyness | The equid rapidly moves its head away from the observer or from other animals. |
| QUESTION 4-29 | 8 - Completely withdrawn/shut down | The equid shows no interest in its surroundings, including the observer, other animals in the environment or the environment itself. This is usually accompanied by a lowered head position and the equid may have partially closed eyes. |
| QUESTION 4-29 | 9 - Other (please specify) | Record any signs that are not already listed using the free text screen. |

Raw, Z., J. B. Rodrigues, K. Rickards, J. Ryding, S. L. Norris, A. Judge, L. M. Kubasiewicz, T. L. Watson, H. Little, B. Hart, R. Sullivan, C. Garrett and F. A. Burden (2020). "Equid Assessment, Research and Scoping (EARS): The Development and Implementation of a New Equid Welfare Assessment and Monitoring Tool." Animals **10**(2).
